# A Rare Presentation of Ameloblastic Carcinoma of the Sinus Cavity and Skull Base

**DOI:** 10.7759/cureus.6265

**Published:** 2019-12-01

**Authors:** Kelly Landeen, William C Spanos, Steven Powell

**Affiliations:** 1 Otolaryngology, Vanderbilt University Medical Center, Nashville, USA; 2 Otolaryngology, University of South Dakota Sanford School of Medicine, Sioux Falls, USA; 3 Oncology, University of South Dakota Sanford School of Medicine, Sioux Falls, USA

**Keywords:** head and neck, odontogenic, sarcoidosis, pregnancy, skull base, malignancy, endonasal, endoscopic endonasal, adjuvant radiation, adjuvant chemotherapy

## Abstract

Ameloblastic carcinoma (AC) is an exceedingly rare odontogenic cancer about which there is limited information in the literature. We present a case of AC originating in the sinus cavity and extending to the skull base in a patient in the first trimester of pregnancy. Diagnostic work up was complicated by this pregnancy, which delayed radiation exposure with imaging. Once scans were obtained, diagnosis was further complicated by the radiographic similarities between possible lung metastases and previously undiagnosed sarcoid nodules. After thorough work up to rule out metastatic disease, the patient was successfully treated with primary surgical resection followed by adjuvant chemoradiation. The patient remained disease free at one year after therapy. This case demonstrates the importance of thorough work up in the diagnosis of AC, and is an opportunity to review the literature and discuss therapeutic methods to treat this rare, aggressive neoplasm.

## Introduction

Ameloblastic carcinoma (AC) is a locally aggressive odontogenic malignancy of embryonic origin. It is most commonly identified in the mandible or maxilla [1]. Less than 120 cases of AC have been reported in the literature. Of these cases, less than five were identified that originated outside the maxilla or mandible [2]. A presentation of AC with concomitant sarcoidosis and a state of pregnancy has not been described in the literature to date. 

## Case presentation

A 27-year-old female with recurrent right-sided sinus symptoms was referred to an otolaryngologist by her primary care provider. She reported symptoms for 4-5 months, including right facial pressure and pain, right eye pressure, nasal discharge, and right nasal dyspnea. There was no nasal bleeding or visual disturbance at the time of presentation. She had been treated with two courses of antibiotics and a trial of saline irrigations with no relief. She was seven weeks pregnant at the time of initial presentation.

Physical examination demonstrated left septal deviation and crusting around the right middle turbinate with visible granulation tissue. Cranial nerves were grossly intact. A flexible nasal endoscopy performed at the clinic revealed a granulomatous lesion filling the right superior nasal vault extending from the middle meatus to the medial septum. A biopsy of the mass was obtained under local anesthesia. Computed tomography (CT) imaging was postponed pending results of the biopsy due to fetal risk in pregnancy from radiation exposure and intravenous contrast.

Differential diagnoses of a granulomatous nasal lesion include infection such as fungal rhinosinusitis; inflammatory causes such as trauma, intranasal cocaine abuse, and chronic granulomatosis; and neoplastic etiologies including inverting papilloma and nasal cancer [3].

**Figure 1:**
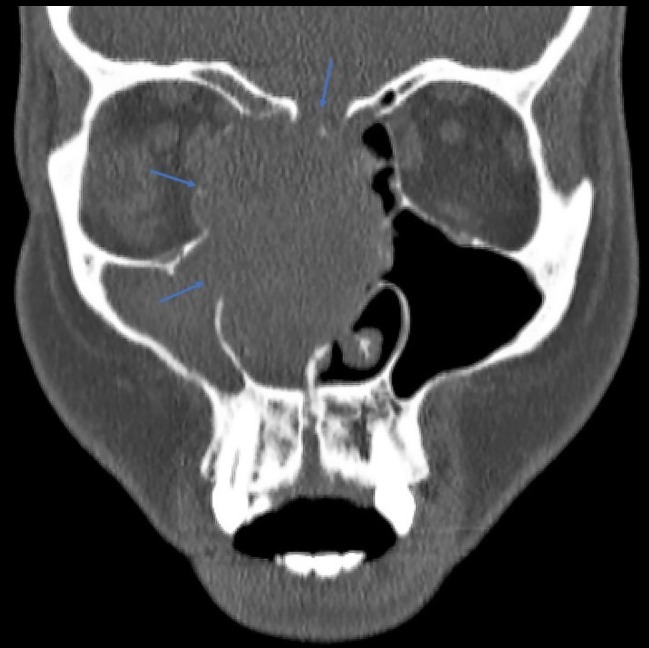
Coronal computed tomography (CT) image demonstrates a 2.6 x 4 x 4.3 cm mass

**Figure 2:**
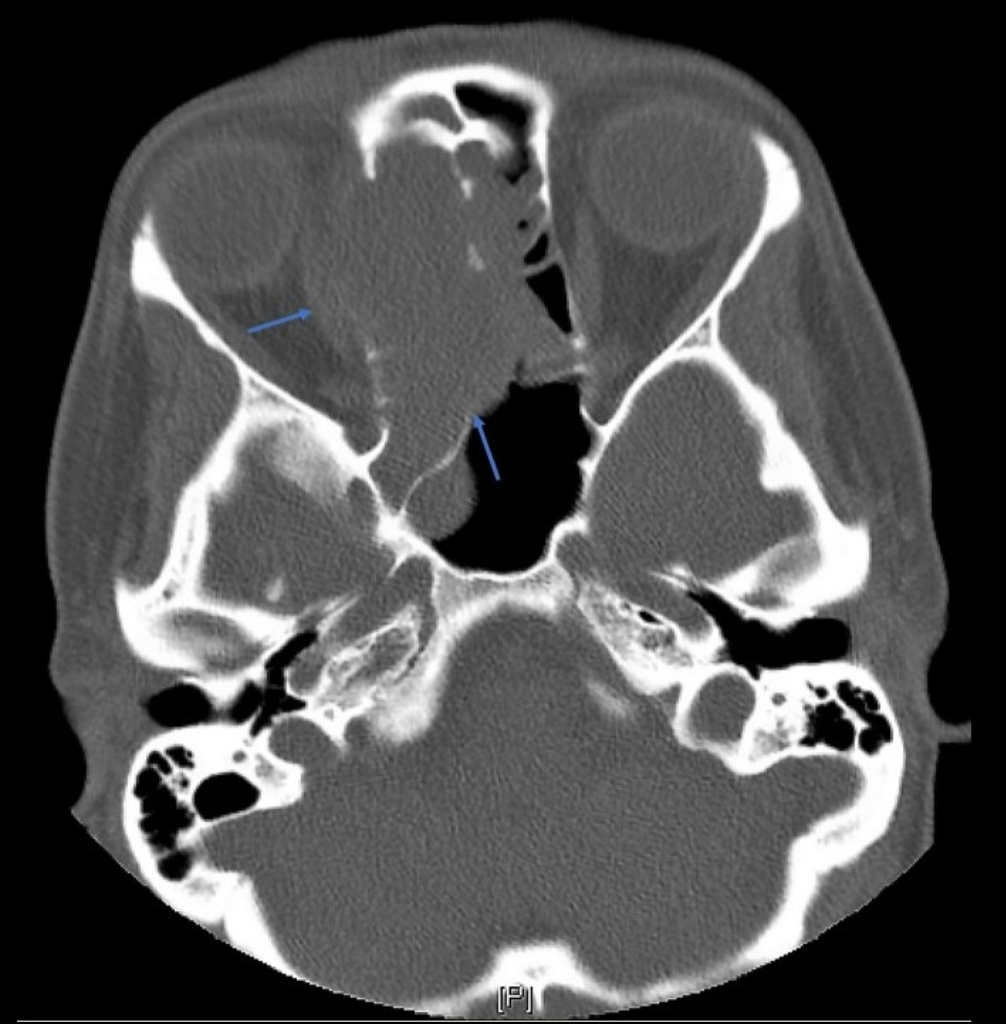
Transverse computed tomography (CT) image demonstrates a 2.6 x 4 x 4.3 cm mass

Pathologic analysis of the patient’s biopsy was suggestive of squamous cell carcinoma in situ, and diagnostic work-up proceeded with non-contrast CT imaging. This demonstrated a large intranasal mass measuring 2.6 x 4 x 4.3 cm, with associated bony destruction in the right medial orbital wall, cribriform plate, lamina papyracea, and planum sphenoidale (Figures 1-2). Multiple secondary biopsies were then taken, and histopathology report demonstrated odontogenic germline epithelium, high mitotic activity, central stellate clearing, and focal p16 immunohistochemical positivity consistent with AC.

**Figure 3:**
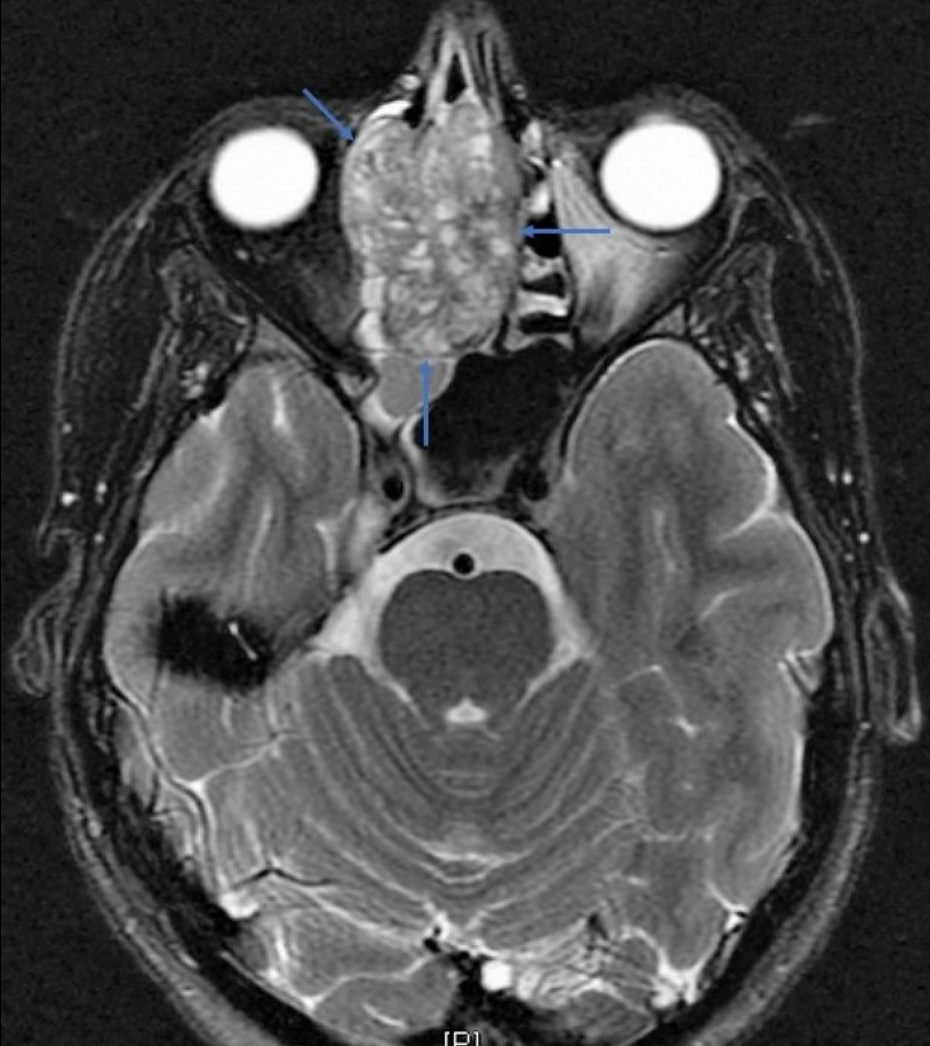
Transverse magnetic resonance imaging (MRI) without contrast confirms computed tomography findings and demonstrates extension of mass into extraconal right orbit

Further imaging was performed with non-contrast magnetic resonance imaging (MRI), which confirmed findings found on CT as well as mucosal thickening of the right maxillary, sphenoid, and frontal sinuses. MRI also showed lateral bowing of the mass into the extraconal compartment of the right orbit, with no definite extension through the periosteum or involvement of extraocular muscles and no clear brain invasion (Figure 3). There were no pathologically enlarged lymph nodes in the head or neck. A 1.6 x 1.6 cm nodule was incidentally identified in the right lung apex, with a possible smaller lung nodule approximately 7 mm in left lung apex, raising suspicion for metastatic disease.

The patient experienced increasing pressure and pain in the nasal and facial regions, as well as proptosis and progressive nasal bleeding. The right nasal passages became completely obstructed within one month of presentation. She had no respiratory symptoms upon initial presentation, but began to develop progressive dyspnea that was present at rest. She did not experience hemoptysis or other respiratory symptoms.

Biopsies of the patient’s lesion were sent for genomic and proteomic profiling through GPS CancerTM (NantHealth, Inc, Culver City, California). This demonstrated normal levels of ERCC1 and TUBB3 markers, indicating that the tumor was sensitive to platinum agents and taxanes. Sensitivity to chemotherapeutic agents in clinical trials targeting MSLN-protein were performed but were inconclusive. No other targetable genes, tumor suppressors, or oncogenes were found.

**Figure 4:**
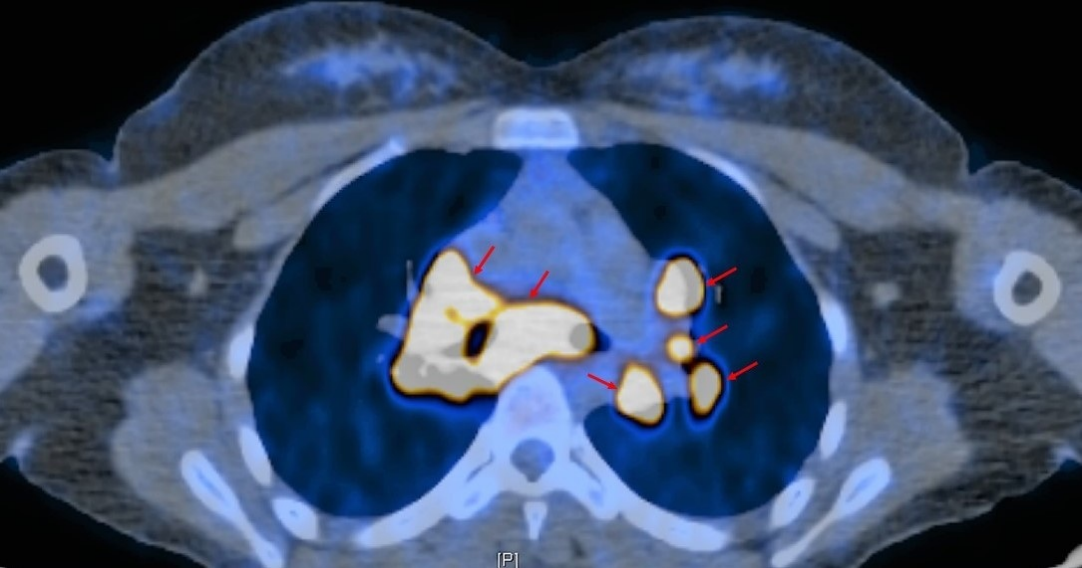
High metabolic activity on positron emission tomography/computed tomography (PET/CT) of the chest is concerning for metastatic disease

**Figure 5:**
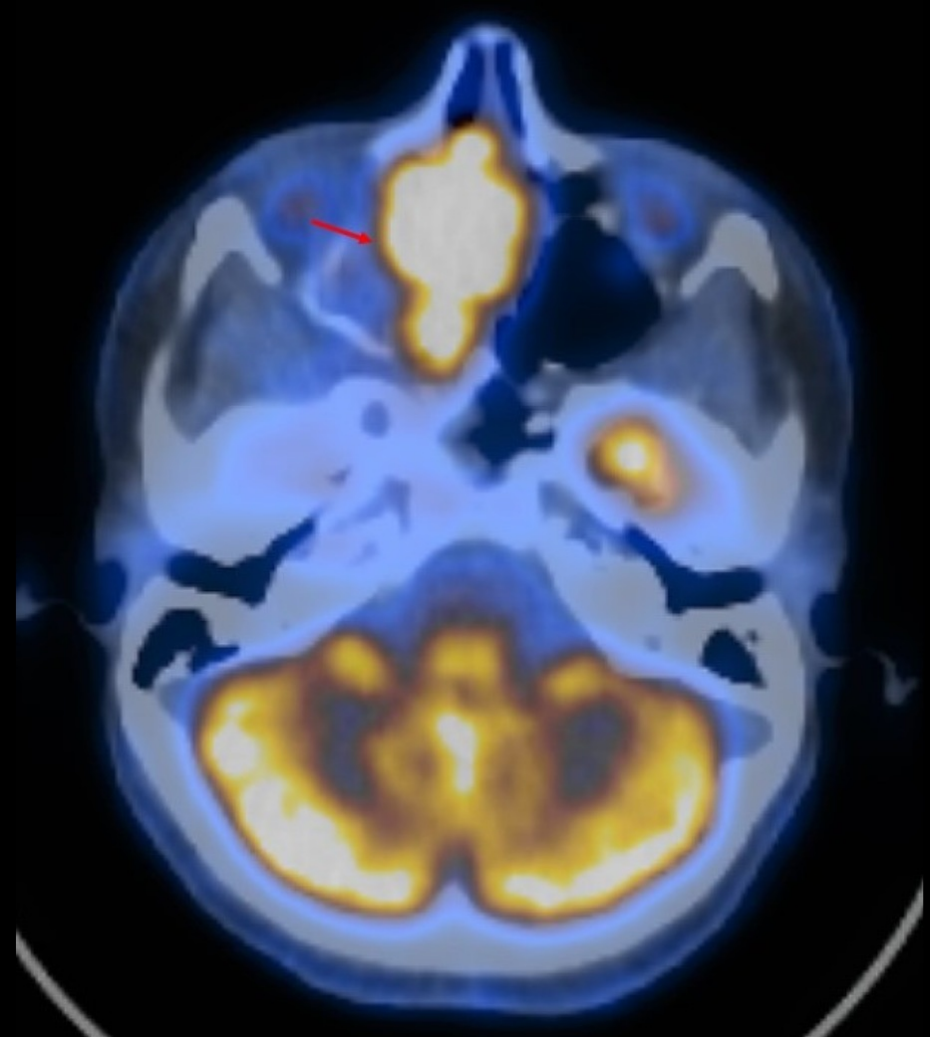
Positron emission tomography/computed tomography (PET/CT) demonstrates high fluorodeoxyglucose (FDG) uptake by the primary neoplasm

A positron emission tomography CT (PET/CT) was performed for further staging and demonstrated enhancement in the right nasal cavity, as well as enlarged fluorodeoxyglucose (FDG)-avid nodal activity in the mediastinum and bilateral hila. There were multiple bilateral FDG-avid pulmonary lesions including a 1.6 x 1.6 cm nodule in the right lung apex and a 7 mm nodule in the left lung apex (Figures 4-5). No intensely FDG-avid cervical nodes were identified. These findings were concerning for metastatic disease, so CT-guided biopsies of lung nodules were performed. This demonstrated inconclusive granulomatous inflammation, so a definitive diagnosis of metastases could not be made at that time despite high clinical and radiologic suspicion. The tumor was therefore classified with the NCCN TNM classification system as T4b N0 MX.

Surgical excision with adjuvant chemoradiation was considered, as well as palliative treatment and comfort care measures. Due to the patient’s poorly defined tumor staging, it was decided that a surgical debulking would be performed with concurrent CT-guided percutaneous biopsies of lung lesions under anesthesia. The patient elected to terminate her pregnancy at 11 weeks gestation due to the risk of metastatic disease and the teratogenic effects of the associated treatments of radiation and chemotherapy.

**Figure 6:**
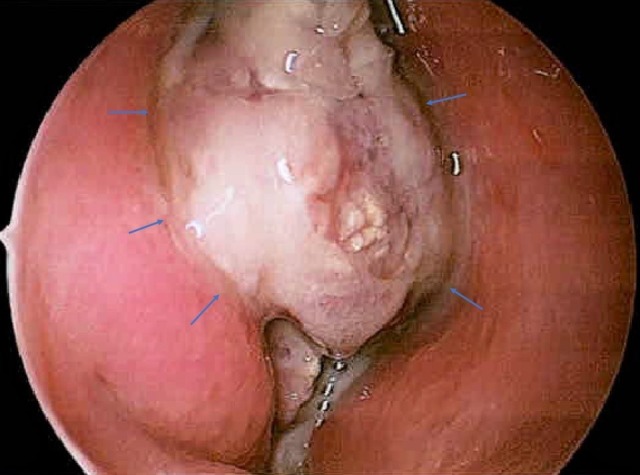
Intraoperative photo of gross tumor extending to middle turbinate

A primary endonasal endoscopic debulking surgery was performed. Tumor was found affixed to the skull base along the cribriform plate and anterior fossa region. The tumor was found to completely obstruct the maxillary antrum and had caused local destruction of the middle turbinate (Figure 6). Biopsies of the mass were taken and the tumor was removed in serial fashion with total estimated debulking of 90%. Simultaneous CT-guided re-biopsy of lung nodules was negative for tumor and consistent with a diagnosis of sarcoidosis. The patient then underwent a second endoscopic surgical resection two weeks later with negative margins on frozen section, indicating complete removal of the primary tumor and no need for dural resection or orbital exenteration.

Due to high risk disease and concern for recurrence, adjuvant chemoradiation was initiated with six cycles of cisplatin (40 mg/m2) and 5580 cGy radiation to the paranasal sinuses. The patient experienced resolution of her initial symptoms, with only minor complications of swelling of the face, likely due to radiation therapy.

**Figure 7:**
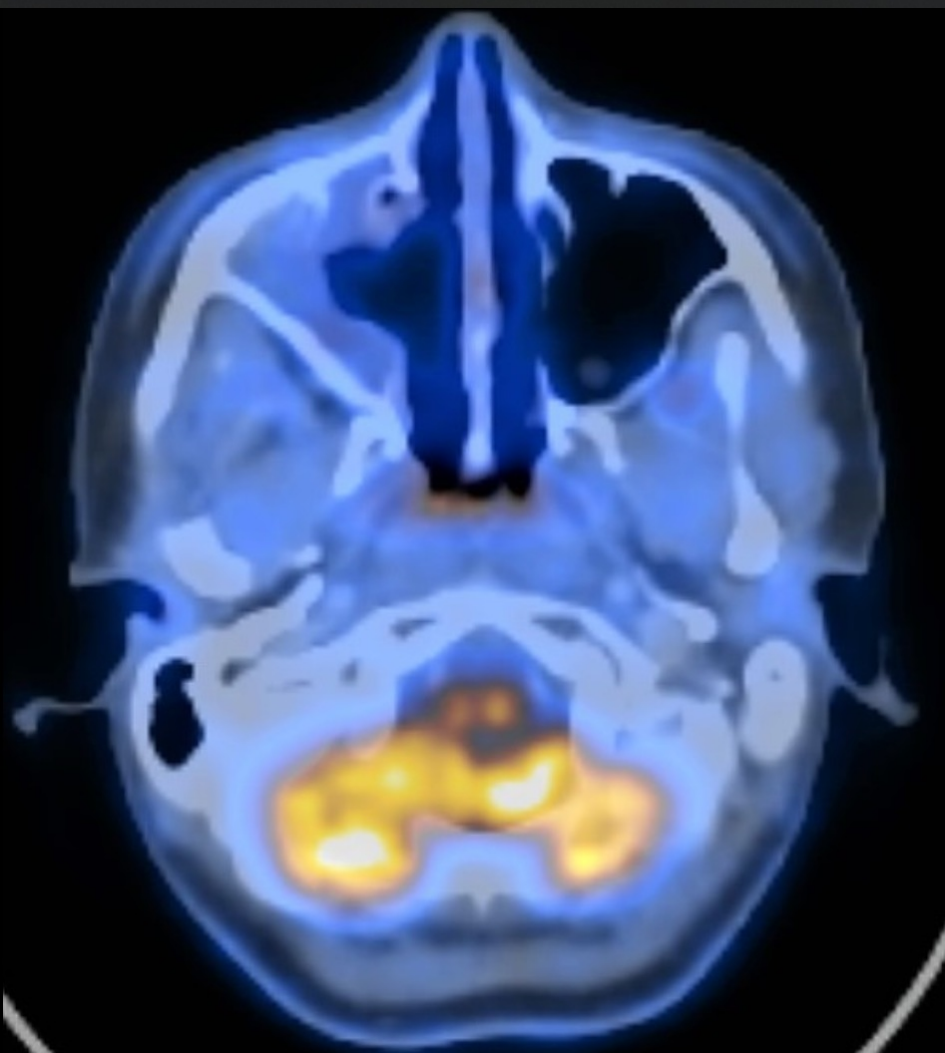
Positron emission tomography/computed tomography (PET/CT) at six months postoperatively shows no signs of recurrent or residual disease

A PET/CT performed six months post-operatively demonstrated no evidence of disease at the primary site and improvement of hypermetabolic adenopathy of the chest (Figure 7). There was new hypermetabolic activity in the abdominal periportal region and the spleen, likely associated with sarcoidosis. Repeat PET/CT imaging done at nine months post-op showed improvement in size and metabolic activity of this new adenopathy, further supporting a diagnosis of waxing and waning sarcoidal inflammation. The patient did not require active treatment for the sarcoidosis and remains on surveillance through the pulmonary service.

## Discussion

The epidemiology of AC is difficult to ascertain due to its extremely low incidence, but one collaborative study found that as of 2016 there were <120 cases of AC reported in the literature [2].

The etiologies of this neoplasm include both primary and secondary AC. Approximately 2% of benign ameloblastomas will metastasize, causing metastatic ameloblastoma or secondary AC. Primary AC is a de novo development of a primary tumor with cytological atypia, including nuclear pleomorphism and high mitotic activity. This classification is made regardless of the presence of metastases at the time of diagnosis. Clinical outcomes are indistinguishable between the two classifications [2,4-7].

The typical clinical symptoms of AC are more aggressive and develop more rapidly than those of ameloblastoma, and include swelling, pain, trismus, dysphonia, expansion of jaws, and perforation of the cortex. These findings are consistent with the initial presentation of this patient. AC metastases spread locally to regional lymph nodes, and can include distant hematogenous spread, most often to the lungs [8].

Initial diagnosis of AC is made with imaging studies. MRI with contrast is the preferred study, although conventional CT with contrast can also be used. However, it can be difficult to distinguish an ameloblastoma from AC with CT and MRI imaging. A more definitive diagnosis of AC can be made with PET/CT imaging and FDG uptake [9]. Definitive diagnosis requires biopsies of the tissue and histopathological confirmation.

This patient’s diagnostic workup was complicated by her pregnancy and sarcoidosis. A CT scan was not performed until after biopsy results suggested carcinoma in order to prevent radiation exposure to the fetus. Similarly, contrast-enhanced imaging with CT and MRI were not used initially due to its potential teratogenic effects. Concurrent sarcoid nodules in the lungs mimicked metastases and complicated disease staging. Sarcoidosis and metastatic disease may present with similar clinical and diagnostic findings, and cases have been reported of sarcoidosis first presenting in the setting of solid malignancy [10]. For this reason, it is important to obtain tissue samples of FDG-avid areas of potential metastatic AC to rule out other causes such as granulomatous disease.

There is no established consensus on treatment of AC due to the low incidence of the disease, although wide local excision is the mainstay of therapy for the primary tumor. As it has been minimally studied, it is unclear if there is a significant difference in cure rates in excision alone versus excision with adjuvant chemoradiation. Regional lymph node dissection is also done selectively and should be considered. Treatment of local extension and metastases usually involves some combination of chemotherapy and radiation. In rare cases, neoadjuvant radiation or chemotherapy is used to shrink a tumor prior to resection. Radiation therapy alone can be effective, but is reserved for local recurrences or cases where the primary tumor may not be surgically resected. In cases of systemic metastases, chemotherapy may be indicated, although studies have demonstrated mixed results. Targeted therapy focused at genomic aberrations is experimental [5,11-13].

The prognosis for a patient with AC is determined by many factors, including tumor size and histopathology. AC carries a more favorable survival rate when compared to other odontogenic malignancies, although the prognosis of AC is usually poor due to advanced disease at the time of presentation. A better outcome is associated with smaller tumor size, younger age at diagnosis, and ability to undergo surgical resection and radiation therapy [7,14]. Approximately 30% of AC cases metastasize prior to diagnosis, and 90% recur after treatment. The overall mortality rate is approximately 30%. Diagnosis at an early stage and routine follow-up with screening for metastases and recurrence are important factors in a patient’s prognosis [2,15].

The existing fund of knowledge regarding AC is limited by the rarity of this disease, and most information is presented in the form of single case reports or case series [16]. This presentation was particularly rare due to the location of the primary neoplasm, which arose from the maxillary sinus near the skull base and involved the cribriform plate area. The case was complicated by sarcoidosis, which mimicked radiologic findings similar to metastatic disease, and by an atypical approach to diagnostic work up and imaging due to the patient’s pregnant state.

## Conclusions

AC is a rare odontogenic malignancy that is often found late in its course and therefore carries a poor prognosis. Due to its low incidence, there is little existing knowledge in its diagnosis and management. Most primary neoplasms are found in the mandible or maxilla; it may arise primarily or as secondary metastasis of an existing ameloblastoma. Diagnosis of AC is best accomplished with radiologic imaging and histopathology, but the diagnostic approach can be complicated by factors such as pregnancy. Due to its propensity to metastasize to the lungs, it is important to obtain pulmonary imaging in any patient with suspected AC. Although a rare malignancy, it is important for clinicians to be aware of the signs and symptoms of AC so that early diagnosis and intervention can be initiated.
